# Effects of KH-204 on the expression of heat shock protein 70 and germ cell apoptosis in infertility rat models

**DOI:** 10.1186/1472-6882-14-367

**Published:** 2014-10-01

**Authors:** Woong Jin Bae, U Syn Ha, Kang Sup Kim, Su Jin Kim, Hyuk Jin Cho, Sung Hoo Hong, Ji Youl Lee, Zhiping Wang, Sung Yeoun Hwang, Sae Woong Kim

**Affiliations:** Catholic Integrative Medicine Research Institute, College of Medicine, The Catholic University of Korea, Seoul, Korea; Department of Urology, College of Medicine, The Catholic University of Korea, 222 Banpo-daero, Seocho-gu, Seoul, 137-701 Republic of Korea; Department of Urology, Second Hospital of Lanzhou University, Lanzhou, China; Korea Bio Medical Science Institute, Seoul, Korea

**Keywords:** Infertility, male, Phytotherapy, Spermatozoa, Antioxidants

## Abstract

**Background:**

Idiopathic infertility is a significant number of causes of male infertility. Empirical treatments are used for idiopathic male infertility, and antioxidant supplementation is a kind of management of oxidative stress related infertility. We investigated the antioxidant effects of the modified Ojayeonjonghwan (KH-204) in a rat model of cryptorchidism.

**Method:**

Male rats were divided into four groups (n = 8 in each): a normal control group, a cryptorchidism-induced control group and two cryptorchidism-induced groups treated p.o. with either 200 or 400 mg/kg, KH-204 for 4 weeks. The testes and epididymides from rats in all groups were removed, weighed and subjected to histological examination and semen analysis after surgery. Oxidative stress was assessed by measuring 8-hydroxy-20-deoxyguanosine (8-OHdG), superoxide dismutase (SOD) and heat shock protein (HSP) levels. Apoptosis was determined using a terminal deoxyribonucleotidyl transferase-mediated dUTP-digoxigenin nick end-labeling assay.

**Results:**

Treatment with the multi-herbal medicine KH-204 (1) increased the mean weight of the cryptorchid testes; (2) restored sperm counts, motility and germinal cell layer thickness; (3) decreased levels of 8-OHdG and increased levels of SOD; and (4) decreased HSP70 levels and apoptosis.

**Conclusions:**

KH-204 reduces the oxidative stress in an experimental rat model of cryptorchidism, and it may alleviate HSP expression and germ cell apoptosis.

## Background

About 15% sexually active couples suffered from infertility and the prevalence rate is increasing [1]. In 40% of these couples, the male partner has been either the single or a contributing cause of infertility [2]. About 42.6% of male infertility is caused by known etiologies such as varicocele, undescended testes, or hypogonadism [3]; however the etiology and pathogenesis are still not fully understood and are referred to as idiopathic infertility in a significant number of cases of male infertility [4]. Currently, empirical treatments are used for idiopathic male infertility, and many pharmacological agents have been used with varying degrees of efficacy. Antioxidant supplement is a kind of management of oxidative stress related infertility, because oxidative stress is one of the main issues associated with male infertility [5]. Several studies have reported that reactive oxygen species (ROS) cause infertility by decreasing sperm motility, breaking the sperm membrane and directly damaging sperm DNA [6, 7].

Several nutritional supplements and herbal medicines have demonstrated antioxidant or free radical scavenging activities [8, 9]. They exhibit beneficial effects on sperm function. For example, Safarinejad et al. showed that nutritional supplement such as selenium, N-acetyl-cysteine, omega-3 and co-enzyme Q10 improved semen parameters [10–12].

Recently, administration of herbs for the treatment of infertility has become popular, despite a lack of scientific studies to assess their effectiveness and safety [13]. Schizandra chinensis Baillon, Rubus coreanus Miquel, Cuscuta chinensis Lam, and Lycium chinense Mill are herbs popularly used in Korean oriental medicine, and these herbal formulation, Ojayeonjonghwan is used to treat male infertility. In addition, each of them has displayed antioxidant activities in a variety of diseases [14–17]. Recently, Kim et al. [18] performed a preliminary study with modified Ojayeonjonghwan investigating the toxicity and influence of five herbs on normal reproductive organs. Their results suggested a safe and positive influence on sperm quality after oral administration of herbal compounds. However, the effects and mechanisms underlying the action of the herbal formulation in an infertility model have not been clearly elucidated. It was hypothesized that the positive influence on sperm quality after treatment would be attributed to the antioxidant effect of each component. In the present study we investigated the antioxidant effects of the herbal formulation KH-204 in an experimental rat model of cryptorchidism.

## Methods

### Preparation of the herbal formula (KH-204)

The major ingredients in the herbal formula were obtained from five plants; 32% Cornus officinalis Sieb. Et Zucc, 4% Schizandra chinensis Baillon, 16% Rubus coreanus Miquel, 16% Cuscuta chinensis Lam, and 32% Lycium chinense Mill. This new herbal formula originated from Korean traditional medicine, and has been shown to be effective for reproductive or erectile function [19]. The content of each plant and the manufacture method are reported by previous studies [18, 19]. Each of the components was placed in 5 L of distilled water and refluxed at 100-120°C for 6 h. The extract was filtered, and the water from the filtrate was removed by a rotary evaporator and a spray dryer (SD-1000, EYELA, Japan). Korea Bio Medical Science Institute (KBMSI) Co. Ltd., a venture company that develops oriental herbal medicines, developed this product as a health supplement.

### Animal groups and treatment protocol

Thirty-two 8-week-old male Sprague–Dawley rats were treated under a protocol approved by the Institutional Animal Care and Use Committee in School of Medicine, The Catholic University of Korea (Approval Nubmer: CUMC-2013-0030-01) and handled according to NIH guidelines. Rats were divided equally into four groups (n = 8 in each): Control, the normal control group; Crypto-control, unilateral cryptorchidism-induced controls; Crypto-200, unilateral cryptorchidism-induced rats administered 200 mg/kg of KH-204; Crypto-400, unilateral cryptorchidism-induced rats administered 400 mg/kg of KH-204. In each group, once-daily oral administration of either distilled water (control, Crypto-control) or KH-204 (Crypto-200, Crypto-400) was continued for 4 weeks. KH-204 was dissolved in distilled water and administered orally through an 8 F red Rob-Nel catheter once a day. The dosage of KH-204 and duration of treatment were selected based on results from a previous experiment [18].

Unilateral cryptorchidism in rats was surgically induced as previously described [20]. Animals were anesthetized with pentobarbital, and a small incision was made in the abdomen of each rat. The left gubernaculum of each rat was cut to displace the left testis into the abdomen, and the left inguinal canal was closed by suturing to prevent testis descent. The right testis, which did not undergo surgery, was used as the paired control. The animals in all groups were killed 4 weeks after surgery, and blood samples were collected. The abdominal control scrotal testes and epididymides were removed and weighed. One-third of the testicular tissue from each individual testis was fixed and embedded for histologic examination, and the remaining tissues were used for measurement of oxidative stress, heat shock protein (HSP) expression and apoptosis.

### Cauda epididymal sperm count and motility

The cauda epididymides were minced in 5 mL normal saline containing 0.5% bovine serum albumin at 37°C and then filtered. Sperm suspensions were placed on glass slides that had been prewarmed to 37°C. The percentage of motile spermatozoa was determined by counting more than 200 spermatozoa in randomly selected fields under a light microscope. Sperm counts were expressed as the number of motile spermatozoa per gram cauda epididymis tissue. An expert investigator blinded to the experimental groups evaluated the samples.

### Histologic evaluation- measurement of spermatogenic cell density

The testicular tissues obtained were fixed in 10% neutral formalin, embedded in paraffin, stained with haematoxylin–eosin and examined under a light microscope at ×400 magnification. Ten representative sites in seminiferous tubules that were almost round were selected randomly and spermatogenic cell density was determined by measuring the thickness of the germinal cell layer and the diameter of the seminiferous tubules.

### Measurement of oxidative stress

Oxidative stress in testes tissues was assessed quantitatively by measuring the 8-hydroxy-2-deoxyguanosine (8-OHdG) and superoxide dismutase (SOD). By use of the DNeasy Blood & Tissue kit (Qiagen, Valencia, CA, USA), total DNA was extracted from the testis. The 8-OHdG levels were measured with a DNA oxidation kit (Highly Sensitive 8-OHdG Check ELISA; Japan Institute for the Control of Aging, Fukuroi, Japan). After the final color was developed with the addition of 3, 3′, 5, 5′-tetramethylbenzidine, absorbance was measured at 450 nm. Tissue sample concentration was calculated from a standard curve and was corrected for DNA concentration. SOD activity (CuZnSOD and Mn SOD) in tissue was measured using a SOD Assay Kit-WST (Dojindo), monitoring the decrease in the rate of superoxide-mediated reduction of nitroblue tetrazolium at 450 nm using a spectrophotometer.

### HSP 70 determination by western blot analysis

The testis tissues were ground to a fine powder in a liquid nitrogen-cooled mortar and pestle. The testis total protein was extracted using a cell lysis buffer (20 mM Tris–HCl pH7.5, 150 mM NaCl, 1 mM Na2EDTA, 1 mM EGTA, 1% Triton, 2.5 mM sodium pyrophosphate, 1 mM β-glycerophosphate, 1 mM Na3VO4, 1 μg/ml leupeptin and 1 mM phenylmethylsulfonly fluoride). Protein extracts were quantified with the BCA Protein Aassay Reagent (Thermo Scientific, Rockford, IL, USA). Quantitative proteins (20 μg) were boiled in loading buffer (62.6 mM Tris–HCl pH6.8, 2% sodium dodecyl sulfate [SDS], 0.01% bromophenol blue, 10% glycerol, and 100 mM DTT). Proteins were loaded per lane and resolved by 10% (HSP-70) SDS-polyacrylamide gel electrophoresis (SDS-PAGE). Proteins were transferred onto Hybond-ECL nitrocellulose membrane (Amersham Biosciences, Germany) and equal protein loading was verified by Ponceau-S staining (Sigma-Aldrich). The membranes were blocked by treatment 5% non-fat milk in Tris-buffered saline containing 0.1% Tween 20 and membranes were probed with anti-HSP-70 antibody (1:1000; Abcam) and anti-β-Actin antibody (1:10000; SIGMA) for internal control as previously described. Immunoreactivation were emitted by using horseradish peroxydase conjugated secondary antibody (Santa Cruz Biotechnology, CA, USA) Densitometric analysis of band intensity was detected by Luminescent Image Analysis System (LAS-3000; FUJIFILM, Japan) and measured using Multi Gauge 3.0 software (FUJI Photo Film, Japan).

### Assessment of apoptosis- TUNEL assay

Testicular tissue sections were rinsed with phosphate-buffered saline (PBS) after blocking with 0.1% Triton X-100 for 5 min. Terminal deoxyribonucleotidyl transferasemediated dUTP–digoxigenin nick end-labelling (TUNEL, ApopTag In Situ Apoptosis Detection Kits; Millipore, MA, USA) detection solution was dropped on to the sections and they were incubated at 37°C in the dark for 1 h. Nuclear staining with DAPI was carried out for 5 min after rinsing with PBS, and the sections were mounted with 50% glycerol after rinsing with PBS. For the control sections, the TUNEL solution was replaced with PBS. The sections were observed under a fluorescence microscope and photographed, and cells from ten randomly selected visual fields were counted to determine the apoptotic index [21].

### Statistical analysis

All data are presented as the mean ± standard deviation. Data were analysed using SPSS for (version 12.0; SPSS Inc., Chicago, IL, USA). Data were evaluated using analysis of variance (ANOVA), with group comparisons made by Scheffe’s test. A p-value less than 0.05 was considered statistically significant.

## Results

### Testis weight

The mean body weights and testicular weights are listed in Table 1. There were no significant differences in body weights and right (contralateral) testicular weights among the groups. The average left testicular weight from the Crypto-control group was significantly lower than that of the Control group (p < 0.05), but was not different from that of the Crypto-200 group. However, the mean weight of the left testes from the Crypto-400 group was significantly greater than that of the Crypto-control group (p < 0.05).

**Table 1:** **The mean body weights and testicular weights after treatment**

	Body weight	Right testicular weight	Left testicular weight
Control	512.66 ± 11.52	1.71 ± 0.11	1.72 ± 0.31
Crypto-control	550.12 ± 20.12	1.84 ± 0.09	0.47 ± 0.06^*^
Crypto-200	533.33 ± 10.19	1.67 ± 0.20	0.57 ± 0.01
Crypto-400	566.67 ± 13.58	1.70 ± 0.09	0.83 ± 0.15^**^

Data show the mean ± s.d., Analysis of variance test, ^*^Significant statistical difference (p < 0.05) compared with the Control group. ^**^Significant statistical difference (p < 0.05) compared with the Crypto-control group.

### Sperm counts and motility

**Figure 1:**
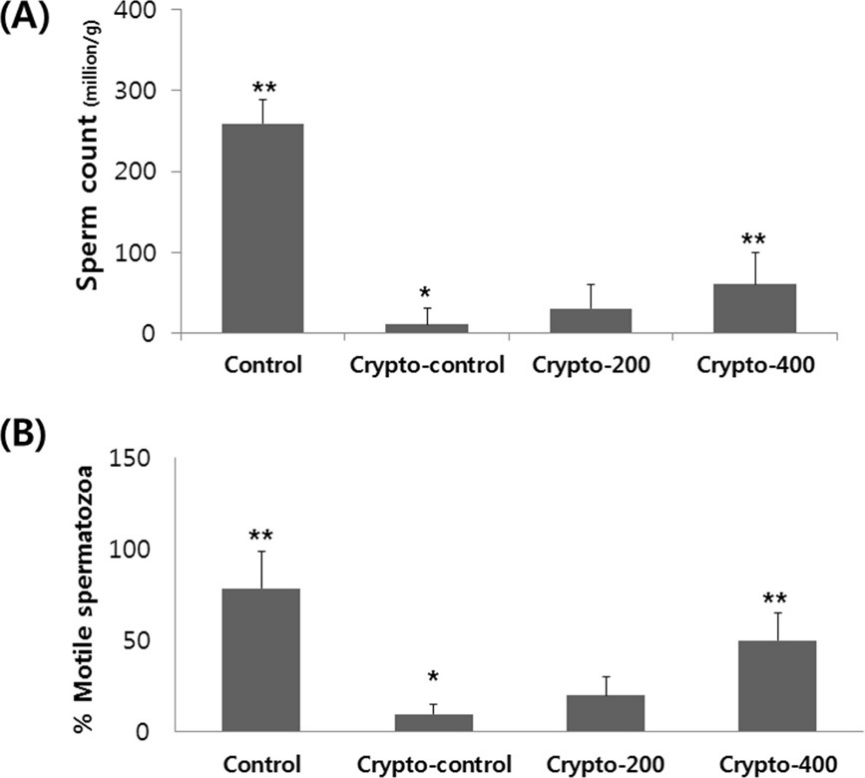
**Effect of the new herbal formula on sperm count and motility.** Sperm count **(A)** and activity **(B)** after 4-week oral administration of the new herbal formula. *, p < 0.05 as compared with the Control group. **, p < 0.05 as compared with the Crypto-control group. By analysis of variance test.

Mean sperm counts and the percentage of motile spermatozoa in the left epididymis are given in Figure 1. The sperm counts and motility from the Crypto-control group were significantly decreased compared with the Control group (p < 0.05), and these same parameters were significantly increased compared with the Crypto-control group after oral administration of 400 mg of the new herbal formula KH-204 (p < 0.05) (Figure 1).

### Spermatogenic cell density

**Figure 2:**
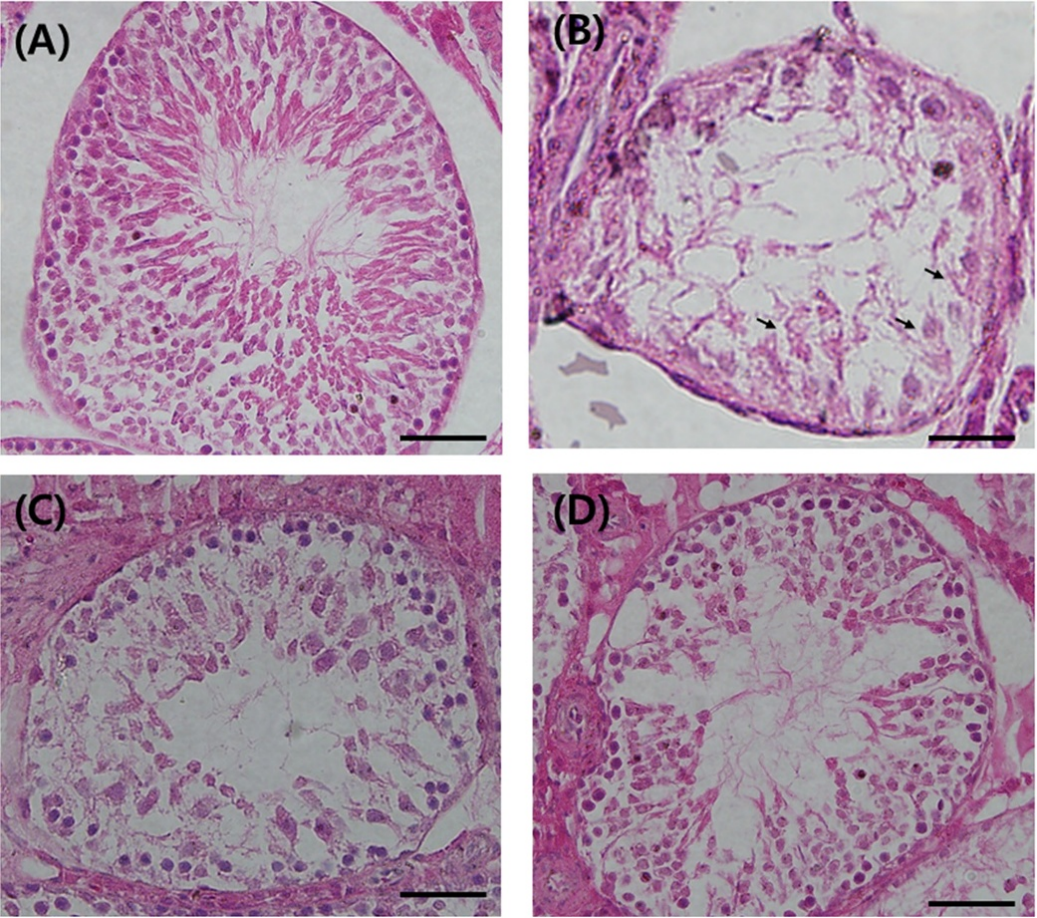
**Histopathological findings of left testis (haematoxylin and eosin stain) in (A) the normal control group (n = 8, Conrtol), (B) the cryptorchidism-induced controls (n = 8, Crypto-control), (C) cryptorchidism-induced rats treated with 200 mg/kg (n = 8, Crypto-200) and (D) cryptorchidism-induced rats treated with 400 mg/kg (n = 8 Crypto-400).** Compared with Conrtol, some necrosis (arrow) and a narrow germinal cell layer is observed Crypto-control. In the treated groups **(C, D)**, the germinal cell layer is thicker than in Crypto-control. Scale bars shown in each figure represent 100 μm.

In the Control group, several layers of spermatocytes formed the germinal cell layer (Figure 2A). However, in the Crypto-control group, the spermatocyte count was decreased, and necrosis was evident (Figure 2B). The spermatocyte count and germinal cell layer thickness were higher in Crypto-200 and Crypto-400 rats than in the Crypto-control (Figure 2C and D). The thickness of the germinal cell layer and the diameter of the seminiferous tubules in the Crypto-400 group was significantly increased compared with that of Crypto-control, and was similar to that of Control rats (Table 2).

**Table 2:** **The thickness of the germinal cell layer and the diameter of the seminiferous tubules in each group (um)**

	Germinal cell layer thickness	Diameter of seminiferous tubules
Control	80.13 ± 5.16	302.25 ± 3.21
Crypto-control	32.38 ± 8.13*	242.48 ± 2.50*
Crypto-200	45.13 ± 1.19	255.71 ± 1.46
Crypto-400	69.23 ± 2.11**	281.54 ± 9.82**

Data show the mean ± s.d., Analysis of variance test, ^*^Significant statistical difference (p < 0.05) compared with the Control group. ^**^Significant statistical difference (p < 0.05) compared with the Crypto-control group.

### Measurement of oxidative stress

**Figure 3:**
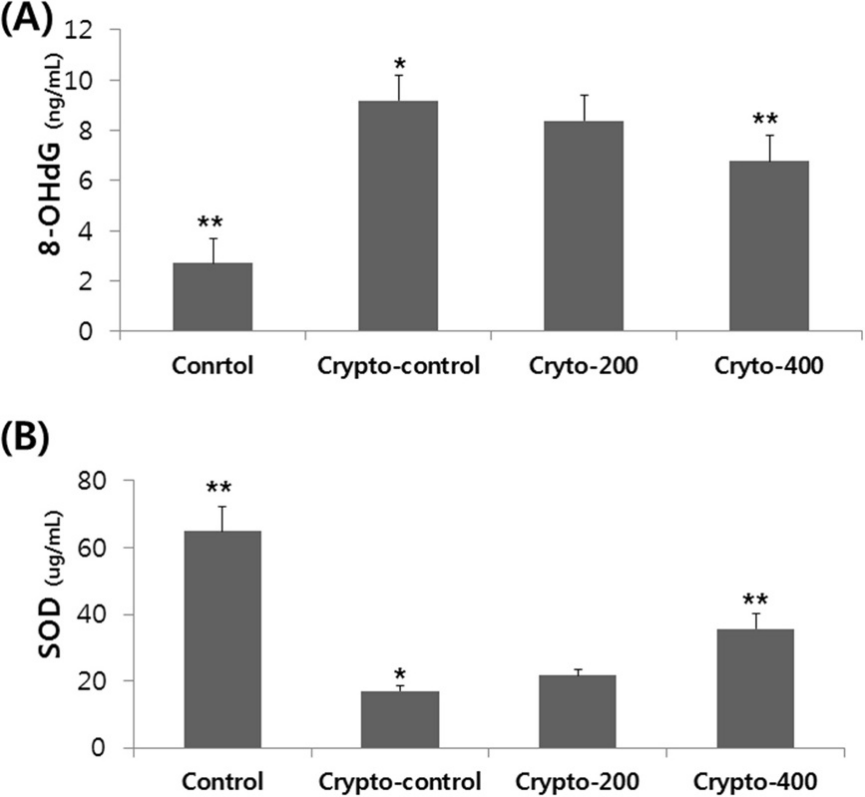
**Comparison of the expression levels of 8-OHdG (A) and SOD (B).** *, p < 0.05 as compared with the Control group. **, p < 0.05 as compared with Crypto-control. By analysis of variance test.

Mean 8-OHdG and SOD expression in the left testes are shown in Figure 3. A dose-dependent decrease in 8-OHdG and an increase in SOD were observed in the treatment groups. Oxidative stress was found to be significantly higher in the Crypto-control group compared with the Control group (p < 0.05), but significantly lower in Crypto-200 and Cryto-400 rats compared with Crypto-control rats (p < 0.05).

### Expression of HSP 70

**Figure 4:**
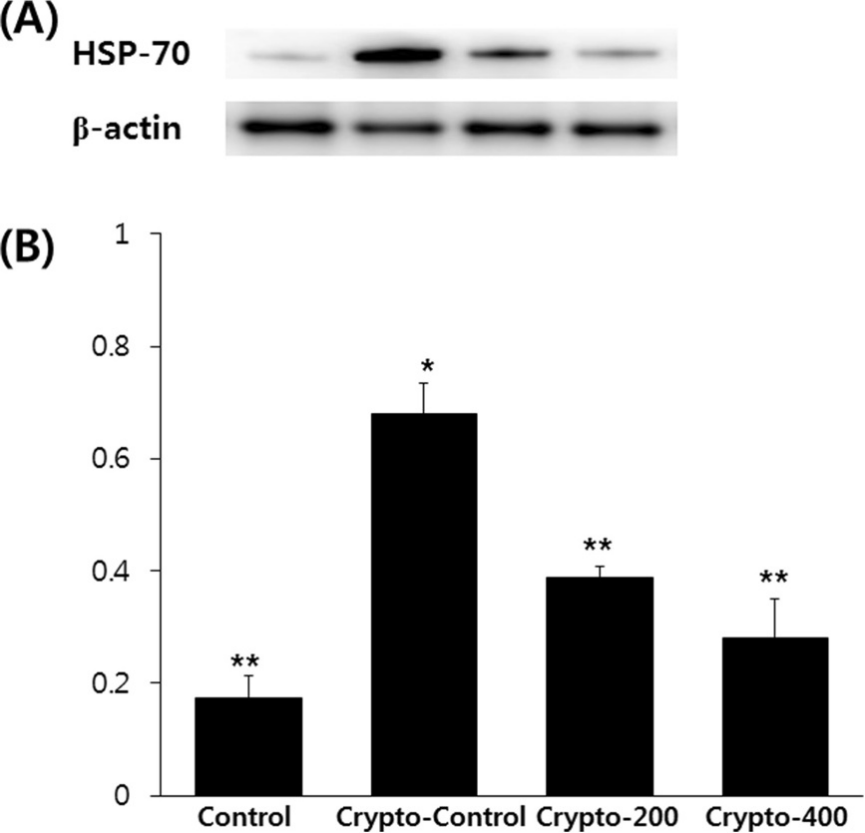
**Comparison of the expression levels of HSP70 (A) and densitometric analysis relative to beta-actin of HSP70 (B).** *, p < 0.05 as compared with the Control group. **, p < 0.05 as compared with the Crypto-control group. By analysis of variance test.

Significant increases in HSP70 were exhibited in the Crypto-control group, but KH-204 administration significantly decreased HSP70 levels in the testes of experimental rats (Figure 4). HSP70 levels in the Crypto-200 and Crypto-400 group were significantly decreased compared with those of the Crypto-control and were similar to those of Control rats.

### Assessment of apoptosis

**Figure 5:**
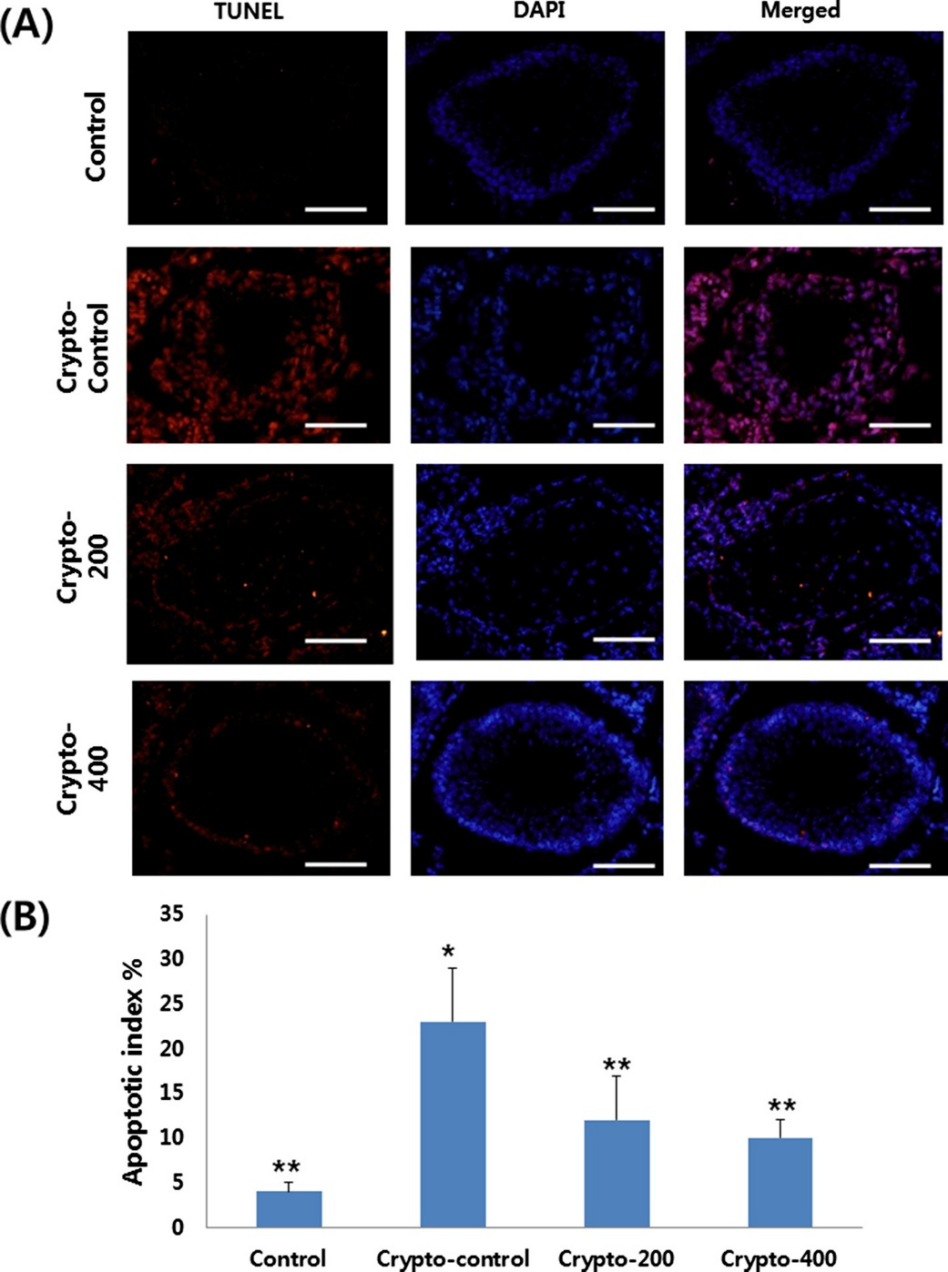
**Effect of KH-204 on testicular tissue apoptosis by TUNEL assay. (A)** Immunofluorescence staining; **(B)** Apoptotic index %. Scale bars shown in each figure represent 100 μm. *, p < 0.05 as compared with the Control group. **, p < 0.05 as compared with the Crypto-control group. By analysis of variance test.

Cells undergoing apoptosis form cellular apoptotic cells, which were observed as dark red in color in the TUNEL assay (Figure 5). Compared with the Crypto-control, the apoptotic cell counts in the left testes were significantly lower in the Control, Crypto-200 and Crypto-400 groups (p < 0.05, respectively).

## Discussion

The main findings of our study were as follows: (1) treatment with the multi-herbal medicine KH-204 increased the mean weight of the cryptorchid testis; (2) treatment with KH-204 restored sperm counts, motility and germinal cell layer thickness; (3) levels of 8-OHdG decreased and levels of SOD increased following treatment with KH-204; and (4) HSP70 levels and apoptotic cells in the treatment group were significantly decreased as compared with those of the Crypto-control group and were similar to those of the Control group.

We observed that the mean volume of the left testes in the cryptorchidism-induced group without treatment was significantly lower than that of the pared control right testes at 4 weeks as well as that of the left testes in the normal control group. In addition, the spermatogenic cell density of the left testes was significantly lower in cryptorchidism-induced controls than in the normal control group. Heat stress induces the generation of ROS in the testes [22, 23] and a reduction of endogenous antioxidant enzymes such as SOD and catalase [24]. SOD plays an important role in the male testes. A recent study demontrated that the presence of high levels of CuZn SOD and Zn render spermatogonia resistant to ROS, and consequently protected from oxidative stress [25]. Superoxide anion, hydroxyl radical, nitric oxide and hydrogen peroxide are among the ROS generated [26, 27]. These ROS are transitory molecules with a high degree of chemical reactivity that could affect spermatogonia for life, thus reducing sperm production and male fertility [28]. Excessive oxidative stress contributes to a deterioration of sperm quality by breaking the balance between ROS and antioxidants in men with infertility. Increased ROS production leads to the peroxidation of the sperm acrosomal membrane and decreased acrosin activity; therefore, the fertilization process cannot be completed [29, 30]. Furthermore, ROS directly damages the sperm DNA by directly attacking the DNA molecule or by initiating apoptosis within the sperm, which induces the caspase-mediated enzymatic degradation of DNA [31]. Therefore, antioxidant administration may help to restore the imbalance of an excessive level of ROS and improve sperm quality. Several oral supplements and herbs have been proposed to improve male factor infertility [32]. These supplements have been shown to instigate improvement in sperm parameters, and the antioxidant properties of these supplements are thought to counteract ROS found in higher concentrations among some men with oligoasthenospermia [10, 33–36].

A number of stimuli induce HSP70, including energy depletion, hypoxia, acidosis, ischemia-reperfusion and ROS [37]. However, the association of HSP70 with semen quality is not clearly understood in experimental animals and humans, and results obtained are conflicting. Rockett et al. [38] demonstrated that both the levels of heat-inducible HSP70 and apoptosis were increased in mice spermatocytes exposed to heat. Their results indicated that under same conditions, spermatogenesis was disrupted. In our study, an increase in the expression of HSP70 was observed in the cryptorchidism-induced controls (Crypto-control) with an increase in free radical exposure. Under conditions of oxidative stress, testicular germ cells can produce high levels of stress response proteins, including HSPs, which protect against free radical insult [39]. Erata et al. [40] evaluated the expression of HSP70 in male infertility and found a positive correlation with DNA damage detected in sperm. It seems to confirm that increased HSP70 expression would assist in blocking aggregation and refolding of damaged proteins as a general protective response. In general, DNA bases and phosphodiester backbones are particularly susceptible to oxidative stress. Many reports suggests that high levels of reactive oxygen species cause the DNA fragmentation commonly observed in the spermatozoa of infertile men [41–43]. Previously also improvements in redox status were associated with reduced HSP70 mRNA levels in rats. In the present study, the decrease in the protein expression of HSP70 was observed in treatment groups.

We found that KH-204 treatment can decrease oxdiative stress and apoptosis in cryptorchidism-induced rats. It is likely that the therapeutic effects of KH-204 are at least in part attributable to suppression of ROS production. Our results may provide relevant evidence for the use of the new herbal formula for treating male infertility. A previous study demonstrated that the toxicity of KH-204 might be less than that of the several herbs such as Rubus coreanus and Cuscuta chinensis when used alone; hence, the safety of this new formulation has been proven to some degree [18].

One potential limitation of our study was that the model used is far from an accurate model of infertility secondary to oxidative stress. It is well known that oxidative stress, which is caused by the excessive generation of ROS, can also induce DNA damage in spermatozoa [44, 45]. Heat stress is implicated in the induction of oxidative stress within the testis [24, 46]. Secondly, through this study, we identified anti-oxidant and anti-apoptotic effects of a new herbal formulation in cryptorchidism-induced rats, however, while we observed improvement, we did not see complete restoration of sperm counts, motility or germinal cell layer thickness. In addition, there is no time-course data although we observed anti-oxidant effect in a dose-dependent manner. Future work should investigate the detailed mechanism of KH-204 with its anti-oxidant effect in subfertility animal model.

## Conclusion

The present study demonstrated that KH-204 reduces the oxidative stress in an experimental rat model of cryptorchidism, and it may alleviate HSP expression and germ cell apoptosis.
